# Using Auxiliary Information to Improve Wildlife Disease Surveillance When Infected Animals Are Not Detected: A Bayesian Approach

**DOI:** 10.1371/journal.pone.0089843

**Published:** 2014-03-27

**Authors:** Dennis M. Heisey, Christopher S. Jennelle, Robin E. Russell, Daniel P. Walsh

**Affiliations:** 1 United States Geological Survey, National Wildlife Health Center, Madison, Wisconsin, United States of America; 2 Department of Forest and Wildlife Ecology, University of Wisconsin, Madison, Wisconsin, United States of America; University of Melbourne, Australia

## Abstract

There are numerous situations in which it is important to determine whether a particular disease of interest is present in a free-ranging wildlife population. However adequate disease surveillance can be labor-intensive and expensive and thus there is substantial motivation to conduct it as efficiently as possible. Surveillance is often based on the assumption of a simple random sample, but this can almost always be improved upon if there is auxiliary information available about disease risk factors. We present a Bayesian approach to disease surveillance when auxiliary risk information is available which will usually allow for substantial improvements over simple random sampling. Others have employed risk weights in surveillance, but this can result in overly optimistic statements regarding freedom from disease due to not accounting for the uncertainty in the auxiliary information; our approach remedies this. We compare our Bayesian approach to a published example of risk weights applied to chronic wasting disease in deer in Colorado, and we also present calculations to examine when uncertainty in the auxiliary information has a serious impact on the risk weights approach. Our approach allows “apples-to-apples” comparisons of surveillance efficiencies between units where heterogeneous samples were collected.

## Introduction

Managing a harmful contagious disease in either domestic animals or wildlife can typically result in expensive and unpopular actions such as culling and quarantine. For free-ranging wildlife populations subject to hunting, restrictions on carcass transportation and hunting over bait are other examples of common but unpopular disease management tools. If a management unit (or population) can be declared “disease-free”, these actions can be avoided. Demonstrating “freedom from disease” in an animal population is rooted in regulatory requirements pertaining to national and international domestic animal trade [1], although the phrase can be conceptually and legally ambiguous [2],[3]. The only way to demonstrate an area is truly disease-free is to conduct a complete census, which is almost always impractical. Instead, a sample of animals from a population must be tested to obtain a probabilistic statement about disease level. Sampling methods to achieve this goal have been investigated for several decades (see [4] for review), and management agencies are increasingly tasked with developing sampling programs in free ranging wildlife [5],[6].

Although the term freedom-from-disease is commonly used in this sampling setting [7],[8],[9], it is a misnomer from the statistical point of view. What is usually actually meant is that disease prevalence *π* is ascertained to be below some designated target threshold *π_t_* with some level of “assurance” (we elaborate on what this means later). Indeed, policy may allow an area to be declared “free from disease” even if disease is observed to be present, but at an acceptably low level
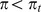
(e.g., [7]). Determining what target prevalence level *π_t_* is acceptably low for an area to be deemed disease-free is a case-by-case policy issue (see [3]) which we do not address; our concern is in efficiently determining whether the true prevalence *π* is less than the target threshold *π_t_* with an appropriate degree of statistical assurance.

Sampling to ascertain freedom-from-disease is often referred to as surveillance. Surveillance in free-ranging wildlife is typically very expensive financially and logistically. While easy to design, popular simple random sampling (SRS) may also be the least efficient for heterogeneous populations and will in general require the greatest number of samples to determine 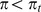
with the degree of prescribed assurance. Large gains over SRS in terms of information per sample can often be realized with some type of stratified, targeted, or weighted sampling.

We use surveillance for chronic wasting disease (CWD) for illustration. Chronic wasting disease is a prion disease of free-ranging and captive cervids (deer, elk, moose), and although there is no evidence that it has been transmitted to humans, it is concerning because prion diseases also include bovine spongiform encephalopathy (BSE), or “mad cow” disease, known to be transmissible to humans. CWD has been discovered in 15 states in the United States of America, and negatively affects cervid populations in those regions. Additionally, its known extent continues to spread [10]. For such diseases, it is desirable to detect their presence while the prevalence is still very low and hopefully amenable to management, hence the target thresholds *π_t_* are typically quite low, and potentially very challenging to assess. We show the value in using what is known about individual-specific infection risk factors obtained from outside the surveillance area but reasonably generalizable to the surveillance area. This work was initially motivated to facilitate the design of efficient surveillance. However, our approach also provides a valuable tool for evaluating the effectiveness of surveillance already conducted; in fact we would argue an approach such as ours is really the only valid way to make “apples-to-apples” comparisons of the effectiveness of multiple surveillance efforts (either in space or time) involving heterogeneous samples.

Several papers have considered the SRS designs of CWD surveillance programs for free-ranging wild animal populations [11],[12]. Nusser et al. [13] explored sampling designs in a simulated CWD system and highlighted the shortcomings of convenience sampling, proposing alternatives based on probability sampling. Diefenbach, Rosenberry and Boyd [14] considered CWD surveillance protocols in Pennsylvania and called for an expanded surveillance stream beyond sole reliance on hunter harvested deer. More recently, to exploit auxiliary information about CWD risk, Walsh and Miller (WM; [15]) developed a surveillance design incorporating a points-based risk factor weighting system (also see [16]). By focusing on high value (high risk) animals, statistical assurance of freedom-from-disease can be achieved with substantially fewer animals than SRS.

Like stratified sampling designs, weighted surveillance programs such as WM's promise great increases in efficiency when there is substantial population heterogeneity in the measured response (e.g. infected or not) associated with observable risk factors. Typically two sample sets are required: 1) a “learning sample” from an area where the disease is present, and 2) the “surveillance sample” from the area to be evaluated. The learning sample is used to determine what factors, such as age and sex, put animals at different risks for infection. In essence, this is a regression problem, where the risk weights are estimated by (potentially transformed) regression coefficients from the learning sample. In contrast to the learning sample, it is typical to not observe any diseased animals in the surveillance sample. But even in the absence of positive animals in the surveillance sample, risk weights from the learning set can increase the assurance that 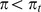
in the surveillance population, which forms the basis of the WM approach. As with regression coefficients in general, the precision and accuracy of these estimated weights depends on the quality and quantity of data used to estimate them. Taking these regression coefficients or weights at face-value without accounting for their uncertainty can result in a considerably over-optimistic assessment of the assurance of 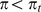.

Some background seems desirable before we present our method. We first present how the goal of disease surveillance is equivalent to applying a confidence or credible bound on *π.* This task has historically been a statistical challenge; when none of *N* samples are positive it amounts to putting a confidence bound on an estimate of zero. Special techniques are required, and there are both frequentist confidence bound and Bayesian credible bound procedures that perform well for the 0/*N* situation. However, because of the nature of the calculations, frequentist confidence bounds appear to troublesome when auxiliary data (a learning set) exists, while Bayesian procedures appear to generalize naturally. Therefore, we propose a Bayesian approach that automatically propagates uncertainty about risk factors and weights sampled individuals appropriately. The development of our approach is as follows:

1. We first show SRS surveillance as traditionally designed and conducted can be productively framed as a traditional one-sided hypothesis test.
2. Then, we note the equivalence of one-sided hypothesis tests and confidence bounds.
3. Frequentist confidence bounds work fine in the SRS context, but Bayesian credible bounds prove more useful for extension to auxiliary information. So, we develop a Bayesian credible bound that has good frequentist coverage properties using a “prior matching” approach. Prior matching involves identifying Bayesian posterior probabilities that also have interpretations as frequentist confidence intervals [17].
4. We briefly consider various concepts of “statistical assurance”; although confidence and credible bounds “look” similar, they have very different substantive interpretations with respect to what they tell us about 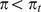.
5. We show how a learning and surveillance sample can be combined into a single augmented regression analysis containing data set-specific intercepts, and how statements about the assurance of 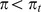 in the surveillance sample can then be formulated in terms of either confidence or credible bounds on the surveillance sample-specific intercept. This is readily accomplished in a Bayesian setting using the software WinBUGS [18], which generalizes the credible bounds from the SRS situation. We present a hypothetical example, and distinguish between what we call “nominal” and “real” weights. The rationale for the specific regression models structure is presented in Appendix S1 (Weight Models).
6. We compare real weights, which accommodate uncertainty in the learning data set, to nominal weights, which are unadjusted point estimates of sample weights. We demonstrate that real weights should be preferred when the learning data set is small to moderate in size. We compare our real and nominal weights to the WM's weights obtained for Colorado mule deer (*Odocoileus hemionus*).
7. We discuss the application of our ideas from both surveillance design and surveillance evaluation perspectives. We advocate real weights as a heuristic surveillance design tool; that is, various sample compositions can be evaluated by totaling up their real weight point scores. To evaluate a surveillance program after it has been performed, we advocate an exact posterior bound approach.

We provide the software code that we used for our examples in the Supplement.

## Model Background

### Freedom-from-disease surveillance as a traditional hypothesis test

Frequentist hypothesis tests are usually designed such that the Type I error rate (*α*) is controlled at some prescribed level. The Type I error rate is the probability that the alternate hypothesis (H_A_) is declared true when in fact the null hypothesis (H_0_) is true. Statistical hypothesis tests should be structured such that the Type I error is the scientifically more important because hypothesis tests generally do not control the Type II error rate [19]. Regardless of whether our focus is on wildlife, domestic animal, or human health, in freedom-from-disease testing, we want to control the probability (*α*) we incorrectly declare units disease-free, especially when the consequences of mistakenly concluding a unit is disease-free are serious. If *π_t_* is the disease-free policy target (or threshold) prevalence, with freedom-from-disease really meaning 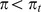, then these considerations lead to the appropriate hypothesis test structure:
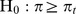





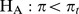



This hypothesis structure reflects that the burden of proof rests on the declaration that a unit is disease-free (e.g., [19], [20], [21]).

Let *C* be the number of positive animals observed in a sample of size *N*. For a declaration of freedom-from-disease to be credible,
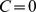
(this might not be the case for all diseases, but few would question this for CWD) prescribes the critical region for this test (probability of rejecting H_0_), which means the total sample size *N* must be manipulated to achieve *α* (this is a little different from most typical testing setups in which *N* is fixed and the critical region is manipulated to achieve *α*, but this is of no particular significance otherwise). The Type I error rate *α* is the maximum probability of observing the critical region 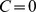
when H_0_: 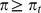
is true. Assuming a large population with SRS, this is generally given as 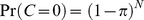
under H_0_, which is maximized at 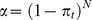. If for example we set 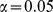
and 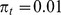, we obtain 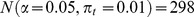 (for small populations, this approach gives conservative results).

These calculations are at the core of most wildlife disease surveillance sampling designs, although they are often not explicitly framed in terms of hypothesis tests. Because the probability of observing at least one positive subject is 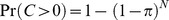, solving 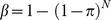 for *N* with fixed *β* and *π_t_* is sometimes described as a surveillance design that achieves a “confidence of *β*” that disease would be detected
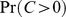
if prevalence is greater than or equal to *π_t_* (e.g., [11]). While this terminology appears to be blending aspects of hypothesis testing and confidence intervals, the intent and effect appear to be essentially the same as our hypothesis test framing.

### The Equivalence of hypothesis tests and confidence bounds

There is a one-to-one correspondence between one-sided hypothesis tests and confidence bounds (e.g., [19]), and an *α*-level one-sided hypothesis test can always be inverted to obtain a 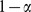
confidence bound (we address confidence intervals/bounds and the notion of coverage probability in more detail later). Thus confidence bounds lead to a unifying interpretation of disease surveillance in terms of supported prevalence values (values consistent with the observation 0/*N*), rather than disease detection in a hypothesis testing framework. Disease surveillance can be reframed in terms of a confidence bound: *N* should be large enough that if 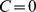 was observed, only prevalence values less than *π_t_* are supported at confidence level 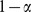. This confidence bound view of disease surveillance is implicit in [21].

Thus, disease surveillance objectives can be viewed “simply” as a problem of constructing narrow enough confidence bounds for the situation when 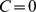
positives out of *N* samples is observed. Although simple in statement, this problem is not so simple in solution. Most confidence intervals involved in the analysis of binary (0–1) data involve large sample theory (asymptotic) approximations. When 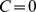, the maximum likelihood estimate (MLE) of prevalence is 0 and the estimate is on the boundary of the parameter space. At this boundary, Brown, Cai and DasGupta ([22]; BCD) show asymptotic approximations break down and a popular solution is to invert the binomial exact test used in the hypothesis test above, referred to as the “exact” or Clopper-Pearson procedure (BCD). Cai [23] and BCD criticize the Clopper-Pearson procedure because it is conservative; it never produces confidence bounds with coverage less than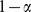, and sometimes coverage can be substantially greater. For freedom-from-disease surveillance, conservative coverage seems reasonable as the method should always perform at least as advertised (e.g., [24]) and we adopt the Clopper-Pearson confidence bound as the standard to emulate as closely as reasonably possible.

### Bayesian Credible Intervals; Reverse Engineering to Achieve Good Coverage

Many Bayesians acknowledge the usefulness of the frequentist notion of confidence interval (or bound) coverage probabilities as a performance metric, and employ it to evaluate Bayesian credible intervals (or bounds). Alternatively, a Bayesian perspective often suggests ways in which frequentist confidence intervals can be improved (e.g., [17], [25]).

As noted by Cai [23], exact binomial Clopper-Pearson confidence bounds correspond to 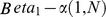, meaning the 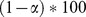 percentile of a
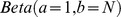
distribution. When viewed in a Bayesian context as a posterior credible bound given the observations 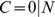, it is reasonable to ask what prior distribution was imposed on *π* to achieve this posterior. To achieve this posterior for a binomial likelihood with 0|*N*, it can be seen that this corresponds to assigning the prior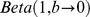 to *π* (Appendix S1; Prior Matching). This is a curiously pessimistic prior that puts all the prior mass at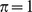; it expresses the prior belief that all animals are infected. This pessimism is consistent with the observed conservatism of Clopper-Pearson bounds. BCD advocate the Jeffreys prior Beta(0.5, 0.5) on the basis that resulting confidence intervals are “more accurate” and “less wasteful” than Clopper-Pearson, but this produces coverage that may be considerably less than 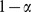 (See [25] for a discussion of Jeffreys and other priors). Our initial analyses indicated that Jeffreys priors can produce coverages substantially less than 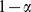
for small *π*, which is a particular problem for freedom-from-disease surveillance studies which should never be liberal. For example, we determined that with
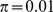
and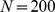, a 95% Jeffreys confidence bound only achieved 87% coverage. We agree with Casella [24] that confidence intervals (and confidence bounds) should perform as advertised; they should never provide substantially less than 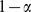
coverage.

A popular “common-sense” prior for the analysis of binary data has long been the Bayes-Laplace prior, which is simply the uniform distribution on 0-1 assigned to *π* and is equivalent to the Beta(1,1) distribution. Tuyl, Gerlac and Mengersen [26] argue strongly for the desirability of this prior based on its predictive properties. The Bayes-Laplace prior leads to what we call the Bayes-Laplace confidence bound, which is easily shown to be 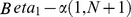 and is equivalent to the Clopper-Pearson confidence bound based on
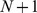
observations (see Appendix S1; Prior Matching). There is essentially no practical difference between the Clopper-Pearson and Bayes-Laplace confidence bounds coverages for moderate sample sizes; in our example above where Jeffreys bounds achieve 87% coverage, Clopper-Pearson and Bayes-Laplace both achieve 100%. While the Bayes-Laplace confidence bound will be slightly more liberal than the Clopper-Pearson, it still tends to be conservative. In a surveillance context, this conservatism is preferable to the liberal error associated with use of Jeffreys prior. We prefer Bayes-Laplace over the “Clopper-Pearson prior” 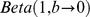, since the latter lacks intuitive motivation and can elicit computational difficulties in the Bayesian MCMC sampling framework (e.g., WinBUGS seems to have problems if *b* is much less than 0.01).

In the Bayesian context, Bayes-Laplace credible bounds inherit the desirable frequentist characteristics of Bayes-Laplace confidence bounds obtained by prior matching. The problem is that confidence bounds for the 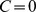 situation seem difficult to generalize beyond the SRS setting, whereas credible bounds seem naturally generalizable, as we will demonstrate. The Bayes-Laplace credible bound is found by solving 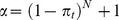, which is easily done by hand calculator. We show how this can also be done in WinBUGS (Software Code S1; Program 1) because it provides a useful stepping stone for later development. If we set 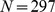, we find the 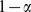 95% percentile of the posterior distribution to be 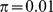, which although practically the same as the Clopper-Pearson confidence bound has a very different interpretation.

### The Two Meanings of Statistical Assurance

Given the importance of confidence and credible bounds to our approach, some background on both is useful. Both impart information about the statistical assurance of parameter values, but the nature of this assurance is very different and worth understanding in the disease surveillance context. It is not uncommon for practitioners to have an unclear understanding of how to interpret a frequentist confidence bound. (Anyone who feels they really understand confidence intervals should read BCD and see if the feeling remains!) A confidence bound is a random variable. A 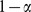
upper confidence bound computed from data as *π_U_* means that in an infinite number of future sets of samples from the same population with true prevalence*π*, if *π_U_* is always computed in the same way in these future samples, in at least
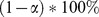
of the infinite future cases 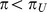
would be true. Thus a confidence bound is a statement about the behavior of a random bound in a series of hypothetical sample sets generated from the same underlying process. The confidence bound generated from a given sample of data can be considered one realization from these sample sets. As such, the frequentist confidence bound provides an indirect, yet intuitive (some would claim), measure of assurance regarding 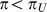
in the sample at hand. Many practitioners erroneously conclude that confidence is simply the probability that 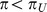, but this is the definition of a Bayesian credible bound [19]. This difference in interpretation reflects the frequentist versus Bayesian interpretation of probability – “the probabilities of things and the probabilities of our beliefs about things” [27].

Confidence bounds can be criticized for the convoluted interpretation that they require when used as a data interpretation tool (e.g., [19]). On the other hand, there seems to be agreement, even among most Bayesians, that the frequentist notion of coverage probability inherent in confidence bounds is a desirable performance metric for assurance measures. The usual criticism of credible bounds is that it requires the subjective specification of prior belief. It could be argued that the ideal assurance measure shares the strong points of both confidence and credible bounds: credible bounds that enjoy the clear Bayesian interpretation yet achieve good coverage behavior when viewed as frequentist confidence bounds. This justifies our use of the Bayes-Laplace confidence/credible bound (see Appendix S1; Prior Matching). However, it is not the clarity of the Bayesian interpretation that primarily motivates our Bayesian approach. With auxiliary data (a learning set), there appears to be no way forward with respect to extending frequentist confidence bounds, while the way is clear with Bayesian credible bounds. We consider this in more detail next.

## Model Implementation

### Changing Scales – Binary Regression Models for Surveillance Data

It is traditional to analyze binary event data, such as disease test data, with logistic regression. In essence, the logistic function remaps prevalence *π* from 0 to 1 to a new scale, the logistic scale, which covers the entire real line. Most software programs allow fitting a regression model without any covariates, that is, an intercept-only, or grand mean model. On the logistic scale, this can be represented as 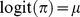, where logit() refers to the logistic transformation of prevalence. If one attempts to fit this model using traditional frequentist logistic regression software for the case where no events 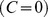 were observed, it will fail because the traditional large sample approximations methods used by popular software break down.

However, the no event case presents no particular challenge in the Bayesian context. This is because Bayesian computations are essentially exact and not based on large sample approximations (WinBUGS uses numerical methods to obtain exact results – the longer it is allowed to run, the smaller the numerical error becomes in general. In contrast, traditional generalized linear model software uses numerical methods to obtain approximate results; numerical errors can be reduced by more iterations but approximation error cannot.)

We illustrate how this is done in WinBUGS (Software Code S1; Program 2) for the surveillance situation where 297 animals were sampled with no positives. We use the complementary log-log (cloglog) link instead of the logistic link, but the basic principle remains the same. We used the logistic link above because readers are much more likely to be familiar with it than the cloglog link, but we develop in the Appendix (Appendix S1; Weight Models) why the cloglog link is better suited to our goals. At this point, it may appear the cloglog transform is simply adding an additional, apparently pointless, step beyond our previous Bayes-Laplace approach. Our reason for presenting this stepping stone becomes clear in the next two sections when we consider auxiliary data from outside the surveillance area.

### Modeling Auxiliary Information

Suppose we have sampled an area known to have CWD; we refer to this as auxiliary information or the learning data set (*L*). Sex is known to be a risk factor for CWD; we will use it for illustration. We will use *x* to indicate sex, with 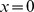
for females and 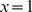
for males. Let *N_x_* be the sex-specific sample size, and let *C_x_* be the respective number of positives found. For illustration, assume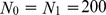,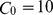, and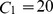; the observed (empirical or apparent) prevalence in males is twice that of females. We could fit the model 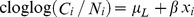, where *i* refers to the *i*-th category of samples. Because *x* is the male indicator, the intercept *μ_L_* is the cloglog-transformed female prevalence in the learning data set. In this model, *β* is referred to as the log hazard ratio, and
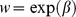
is the hazard ratio (Appendix S1). We will refer to *w* and estimates of it as “nominal weights”.

This model can be readily fitted to the auxiliary data using traditional generalized linear model software. This model is also easily fitted with WinBUGS (Software Code S1; Program 3). (The large-variance normal prior on *β* is a traditional approach to establishing an essentially vague yet proper prior; see for example Appendix C of [28].) For our hypothetical example the posterior mean (and standard deviation) for the male log hazard ratio *β* is 0.656 (0.379). The posterior mean for the hazard ratio 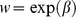, or nominal weight, is 2.07; very close to the observed 2:1 prevalence ratio observed in the data. As noted, the analyses up to this point could be done with either traditional frequentist or Bayesian software. The problem with the frequentist approach would arise when one then attempts to integrate surveillance data in which no positives were observed.

When we obtain a point estimate of the nominal weight *w* our certainty in it will depend on the strength of the learning set, just as for any regression coefficient in general. If our data set is small and we obtain an estimate of *w* of 2, we may be reluctant to conclude that a male is actually worth 2 females, but our certainty in this should increase as the sample size increases. We later describe how to calculate modified weights (which we refer to as real weights) that adjust for such uncertainty due to sample size.

### Augmented Surveillance

We now combine our hypothetical surveillance sample of 297 animals and our learning sample from before. Will the learning data set change our conclusions about surveillance? As we will see, the answer is that it depends. Let *δ_i_* be an indicator variable for whether the *i*-th sample is from the surveillance 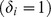
or learning data set 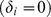. The model for the augmented analysis then becomes 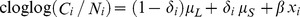, where *μ_L_* and *μ_S_* are data-set specific female intercept terms for the learning and surveillance data sets respectively. Note that we can back-transform *μ_S_* to *π_S_*, the prevalence in the surveillance sample. Given how the model is parameterized, this is the female prevalence, which we have established as the baseline.

Suppose all 297 surveillance subjects are females. If we run this analysis (Software Code S1; Program 4) and establish a 95% credible bound for *π_S_*, we find it is *π_S_* = 0.01. This is exactly as it was before in the absence of any auxiliary learning data. This is exactly as it should be. In the auxiliary analysis, the baseline was established as the females. As the reference group, the observation of a female provides a unit amount of information in the surveillance sample.

Now suppose all 297 surveillance subjects are males (Software Code S1; Program 5). We now find the 95% credible bound for *π_S_* to be 0.0058. Recall that *π_S_* is the prevalence for the baseline female group; we can now be 95% certain that female prevalence *π_S_* is less than 0.0058 with 297 male samples in contrast to 0.01 with 297 female samples. Because the auxiliary sample showed males have a higher risk of infection than the female reference class, they contain more information regarding freedom-from-disease, and hence the smaller bound.

If these models were attempted with traditional maximum likelihood software, they would fail because the MLE for *μ_S_* in the absence of any positive surveillance events is negative infinity, on the boundary of parameter space. Even if this is “patched” by assigning some small number, usual methods for estimating the standard error of the estimate of *μ_S_* will fail. (Technically speaking, this failure results from a violation of what are called the “usual regularity conditions” on which traditional standard error approximations are based – in particular the parameter estimate is not in the interior of the parameter space as required.).

Returning to our augmented analysis, the nominal weight of 2.07 suggests that 1 male is worth a little more than 2 females. But this assessment ignores statistical uncertainty in the nominal weight estimate. We can account for this uncertainty by progressively decreasing the surveillance sample of males until the 95% credible bound matches the target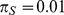, which occurs at about 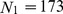
males. We call this approach “target bound-matching”. Thus, a male surveillance sample is worth about
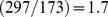
females, given the information, albeit imperfect, from our learning data set. We will refer to weights obtained in this manner, by target bound-matching, as real weights.

## Model Results

### Weights in General

**Figure 1:**
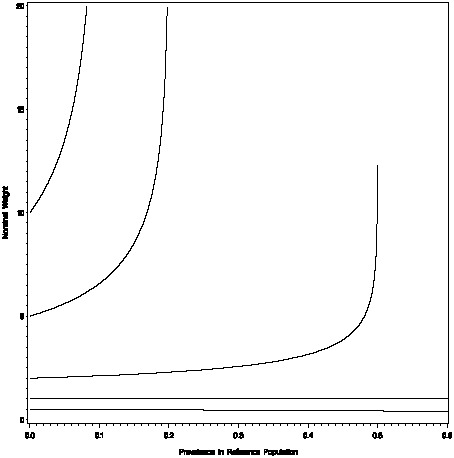
Nominal weights as a function of the prevalence ratio 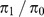
and prevalence *π_0_*. Nominal weights are shown for 5 fixed prevalence ratios: 10, 5, 2, 1, and 0.5, which are in ascending order in the figure. The x-axis is the denominator prevalence *π_0_*. Nominal weights increase rapidly as the numerator prevalence *π_1_* approaches 1; as the numerator class becomes more like a “perfect sentinel”.

WM proposed the idea of weights or “points” that could be assigned to an animal to reflect its value for detecting disease, relative to some reference class of animals for which the point value was 1. Under WM's derivation, the parametric expression of their weight was 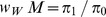 (considering just two classes, with class 0 being the reference class). Under our derivation (Appendix S1) the parametric expression for our (nominal) weight is 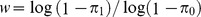. Generally speaking, our derivation of *w* reflects how sample sizes and prevalences influence the probability of detecting at least 1 positive subject in a sample. The different derivations of *w* versus *w_WM_* lead to different behaviors. Because *w* is based on the probability of detecting at least one positive, 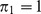
has special significance because it means if even one class 1 animal is sampled, a positive will be detected with certainty. When 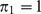, we say that class 1 constitutes a “perfect sentinel” (only one sample is needed), and in which case 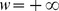
which is arguably desirable behavior (regardless of the prevalence of the reference class, only one perfect sentinel needs to be sampled). Figure 1 displays the parametric relationship between *w* and *w_WM_*, and in particular it shows how they depart as 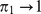.

These are parametric expressions; in reality one uses estimates of these quantities. As these parametric expressions suggest, the quality of the weight estimates rest largely on the quality of the prevalence estimates. If the prevalence estimates are of low quality with substantial uncertainty, estimates of *w* and *w_WM_* should not be taken at face value but should be discounted in some way for estimation uncertainty, which is considered next.

### Nominal Weights versus Real Weights

**Figure 2:**
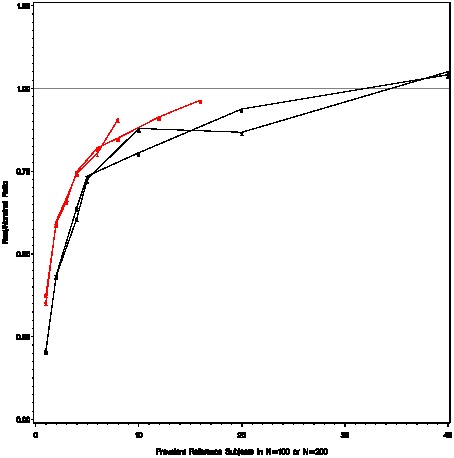
Factors controlling the departure of real and nominal weights. The red curves correspond to a prevalence ratio of 10, and the black curves correspond to a prevalence ratio of 2. For each fixed prevalence ratio, two sample sizes 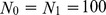
(plotting symbol  = 1) and 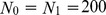
(plotting symbol  = 2) are shown. For a fixed prevalence ratio and sample size, one can vary the number of positives in class 0 (*C_0_*), and compute the corresponding number of positives in class 1 (*C_1_*). The x-axis is *C_0_*. One can then compute the nominal and real weights from *C_0_*, *C_1_*, *N_0_*, and *N_1_*. The primary determinate for departures between the real and nominal weights appears to be the number of positives in the sample (x-axis), and not the total sample size (1 versus 2 plotting symbol). The apparent prevalence ratio (red versus black) appears to play a minor secondary role.

As illustrated previously, we propose a method to obtain weights that are adjusted for uncertainty through a process we call “target bound-matching”, we refer to these uncertainty adjusted weights as “real weights”. For large data sets with many positive cases, real weights and nominal weights should correspond. Here we are primarily interested in the factors that cause them to not correspond, that is, what aspects of an auxiliary data set gives rise to uncertainty when it comes to estimating nominal weights? Considering just two classes, there are 4 statistics involved in estimating weights, which are *N_0_*, *N_1_*, *C_0_*, and *C_1_*: the sample sizes in the two classes, and the number of positives in the two classes, respectively. In this 4-dimensional statistics space, what factors result in large departures between real and nominal weights? This requires exploring this sample space in a systematic manner; we were interested in exploring regions of the space that correspond with potential real world scenarios. One factor we wanted to examine was the role of the empirical prevalence ratio, 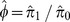, where 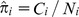. Thus we did two sets of analyses, one for 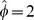 and one for 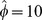. Within each observed prevalence ratio we looked at the effect of total sample size for sample sizes of 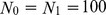 and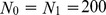. With 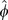
and *N_i_* fixed, it was an easy matter to compute all values of *C_i_* consistent with the fixed 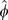
and *N_i_*, which we did. We then compute the corresponding estimated nominal weights 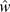
and real weights *R* which we expressed as the ratio 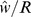 for standardization; the results surprised us somewhat (Figure 2).

The observed prevalence ratio and total sample size has relatively little influence over the estimated nominal weight/real weight ratio. The primary determinate seemed to the number of events (positives) *C_i_*, while the number of trials *N_i_* needed to observe these events seem to have little influence. Figure 2 also clearly illustrates that there is a point of diminishing returns, where the real weights get very close to the nominal weights, and relatively little is learned with additional sampling. The practical implication for the learning set appears to be that one should seek a happy medium; if few positives are observed, the real weights will be low, but beyond a certain number of positives the additional gain in real weight is negligible.

### Real Data Application

Using the auxiliary data from WM's Table 1 of Colorado mule deer/CWD data[15], we first compute point estimates for weights that are not adjusted for uncertainty. We do this in three ways. We used SAS PROC GENMOD (Software Code S1; Program 8) under WM's Poisson assumption to obtain MLEs of their weights *w_WM_*. As we note above, we need to use a Bayesian approach for augmented (surveillance and auxiliary combined) analyses, but the auxiliary data alone can be equally well analyzed by either Bayesian or frequentist methods, and it is interesting to do so for comparative purposes. We computed Bayesian nominal weights for our binomial model (Software Code S1; Program 6) and their maximum likelihood equivalents using SAS PROC GENMOD (Software Code S1; Program 8). As expected, our estimated nominal weights are higher than estimated WM's weights in high prevalence situations (Table 1) because of the sort of behavior demonstrated in Figure 1. The Bayesian and frequentist estimates of our nominal weights are the same for practical purposes.

**Table 1:** Estimates of nominal CWD surveillance weights for 8 classes of mule deer from Colorado (data from WM[15]) using a binomial complementary log-log regression model with Bayesian and maximum likelihood approaches, as well as a Poisson regression model.

	Binomial		Poisson	C/N
Mortality Source	Bayesian (SD)	MLE (SD)	MLE (SD)	
*Suspect-F*	14.1 (2.40)	14.1 (2.4)	11.6 (1.6)	40/111
*Suspect-M*	12.2 (2.05)	12.2 (2.06)	10.3 (1.46)	40/125
*Other*	1.9 (0.24)	1.9 (0.25)	1.9 (0.24)	77/1,300
*Harvest-adult-M*	1 (NA)	1 (NA)	1 (NA)	313/10,046
*Harvest-adult-F*	0.57 (0.06)	0.57 (0.06)	0.58 (0.06)	104/5,782
*Harvest-juv-F*	0.44 (0.15)	0.44 (0.15)	0.45 (0.15)	9/645
*Harvest-juv-M*	0.25 (0.08)	0.25 (0.08)	0.25 (0.08)	11/1,329
*Harvest-fawn*	0.03 (0.03)	0. 03 (0.03)	0.03 (0.03)	1/999

*Notes*: The *Harvest-adult-M* category is used as the reference class in these analyses, as in WM [15]. We provide both the count of CWD positive animals (*C*) and the total number sampled (*N*) from WM [15].

We then used credible bound matching in our Bayesian model to obtain real weights for the Colorado data set (Table 2). The real weights closely matched the estimated nominal weights obtained from the learning data for all but the three lowest weighted classes. This stability reflects the relatively large number of positives in most of the learning set categories.

**Table 2:** Nominal and real surveillance weights calculated using data from WM[15].

Mortality Source	Nominal Weight (SD)	Real Weight (*R_i_*)	*C*/*N*
*Suspect-female*	14.1 (2.40)	13.5	40/111
*Suspect-male*	12.2 (2.05)	11.9	40/125
*Other*	1.9 (0.24)	1.9	77/1,300
*Harvest-adult male*	1 (NA)	1	313/10,046
*Harvest-adult female*	0.57 (0.06)	0.57	104/5,782
*Harvest-yearling female*	0.44 (0.15)	0.39	9/645
*Harvest-yearling male*	0.25 (0.07)	0.23	11/1,392
*Harvest-fawn*	0.03 (0.03)	0.006	1/999

For real weights, a sample equivalent to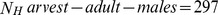 reference class animals was needed to obtain the target goal, which is for the posterior probability 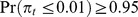.

*Notes*: Values for nominal weights are the Bayesian posterior means of the hazard ratios. Real weights were obtained by posterior credible bound matching, described in the text.

## Discussion

We think real weights are useful to motivate the notion that targeted surveillance schemes should accommodate uncertainty. We also think they are a useful design tool for researchers tasked with conducting surveillance – if testing resources are limited, real weights can be used as a rule-of-thumb to prioritize samples during the design and implementation phases. For example, using the Colorado data for illustration, if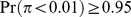 is the goal, and harvested-adult-males are deemed a reasonable reference category for*π*, this goal can be achieved with a surveillance sample of 297 harvested-adult-males. But this goal could also be achieved with as few as 22 suspect-females (297/13.5). In many cases, it may not be possible to simply sample the highest weight subjects, but one could still focus on generally high weight subjects. For example, if one summed up the real weights for 10 suspect-females, 10 suspect-males, and 23 others, the summed real weight is 297.7. When we calculate the exact posterior credible bound for such an observation (Software Code S1; Program 7), we observe 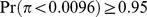, so we have slightly overshot our goal (which is usually not a bad thing).

As this illustrates, we have noticed that in general real weights do not exhibit perfect additivity, in particular when the discrepancy between real and nominal weights is large (not illustrated). Thus, although we feel that real weights are a useful design tool for planning and prioritizing samples and “getting in the ballpark” with respect to the effective sample size, we advocate exact posterior bounds as we just illustrated to evaluate the specific performance of a surveillance sample.

Typically, logistics constrain the number of surveillance samples that a monitoring agency can obtain. Real weights allow the agency to realistically prioritize high-value animals. By summing weights, they can also keep track of how well a surveillance program is progressing by summarizing the “effective” sample size in terms of a synthetic sample of all reference animals; however we recommend some caution in this respect. As we note, real weights are not purely additive, and we advocate that exact posterior bounds be used to rigorously determine whether the surveillance goal of
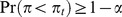
has been achieved. Summed real weights should be viewed as rules-of-thumb, and should be useful in designing and monitoring a surveillance effort.

The choice of reference class is mainly a matter of common sense; it should be some class of animals in which one would be interested in statements about disease presence, typically a common class. This notion is sometimes criticized for its apparent arbitrariness; but this completely misses the point – such criticism is akin a saying the Celsius scale measures temperature better than Fahrenheit. Indeed, without such standardization, it seems essentially impossible to make meaningful comparative statements about how well surveillance has been performed in various units. Clearly, a surveillance sample of 297 harvest-fawns is not equivalent to a sample of 297 harvest-adult-males; only an approach such as ours allows an “apples-to-apples” comparison of certainty of “freedom-from-disease” from different areas.

For CWD, the standard USDA certified tests are regarded as being essentially 100% specific. However, for an animal that has just recently experienced an infection event, it may not test positive, so in this sense the test may not be 100% sensitive, but it is very difficult to quantify this. The most straightforward approach would be to recognize the possibility that apparent prevalence might be lower than true prevalence, and set an appropriately conservative (small) threshold for *π_t_*. If sensitivity and specificity can be quantified, extensions similar to Cameron and Baldock [21] could be explored. Another potential modification would be to include a finite population correction factor if the population size is known [21]; our approach is conservative for small populations, that is, the actual assurance level will probably be higher than the nominal 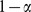
level, which is appropriate for disease surveillance.

Ideally, we recommend agencies collect learning data from their unique disease systems for subsequent calculation of real weights in their surveillance efforts. If relatively local data are available, the desirability of including spatial structure as a risk factor, similar to that employed for disease risk mapping [29], could be explored. If relatively local data are not available, caution should be exercised when generalizing information from other systems. As we presented our approach, we always included the data from the learning set directly into the augmented analysis of the surveillance data. However, we could have partitioned our analyses into two steps – first obtain the posterior distributions for the log hazards from the learning set, and then use these posteriors as priors in the evaluation of the surveillance set, perhaps using normal approximations. If we have doubts about the generalizability of the learning set to our surveillance set, we could manipulate the priors going into the surveillance analysis from the learning set to reflect this added uncertainty, for example increasing their variance to evaluate the sensitivity of the results to this added uncertainty. This is a natural application of the Bayesian concept of uncertainty.

## Supporting Information

Appendix S1 **Prior Matching and Weight Models.** The technical details behind prior matching and our derivation of sample weight models is given here.
(DOC)

Software Code S1 **Software Code for Examples.** The WinBUGS and SAS code is given here for all of our examples.
(DOC)
