# Supplementary material for: Using Auxiliary Information to Improve Wildlife Disease Surveillance When Infected Animals Are Not Detected: A Bayesian Approach
Source: PLoS One. 2014 Mar 27;9(3):e89843. doi: 10.1371/journal.pone.0089843 (PMC3968012; doi:10.1371/journal.pone.0089843)
Supplement: Appendix S1 — Prior Matching and Weight Models. The technical details behind prior matching and our derivation of sample weight models is given here. (DOC) [file pone.0089843.s001.doc]

**Appendix S1**

**Prior Matching**

Suppose the prior density on *π* is $\pi\sim f\left(a,b\right)$, where$f\left(x,y\right)$ is the beta probability density , where$B\left(x,y\right)$ is the beta function. Suppose *C* failures out of *N* trials are observed. From well-known results, the posterior density of *π* is then

$\pi\sim f\left(a+C,b+N-C\right)$. If $C=0$, the $1-\alpha$level upper credible bound on *π* is then the solution to$1-\alpha=\int_{0}^{\pi} f\left(a,b+N\right)dp$ . To perform prior-matching, we want the values of *a* and *b* that solve , where the left-hand-side is the Clopper-Pearson bound. It is elementary to show that , where *c* is some appropriately chosen normalizing constant. This corresponds with the matching beta prior$f\left(a=1,b\to0\right)$ . If we use the Bayes-Laplace prior $f\left(1,1\right)$, we obtain , which is the Clopper-Pearson bound which would result from adding 1 additional success observation, thus the Bayes-Laplace prior will give very slightly more liberal bounds for moderate to large *N* than the Clopper-Pearson matched prior, the latter being known to be conservative, sometimes highly so. It is useful to compare these results through Laplace’s rule of succession, which is the probability of observing a failure on the next $\left(N+1\right)\text{th}$trial given a previous run of *N* successes. For the Bayes-Laplace prior, this probability is $1/\left(N+2\right)$, while for the Clopper-Pearson matched prior it is $1/\left(N+1\right)$. If $N=298$, this gives a Bayes-Laplace succession probability of 0.003333 versus a Clopper-Pearson probability of 0.003344.

**Weight Models**

We start with what we call the “weight postulate”. The weight postulate essentially states that the concept of nominal sample weights is a reasonable one. Consider two groups 1 and 2, with prevalences *π_1_* and *π_2_*. The weight postulate says that it is reasonable to equate the information about *π_1_* in a sample of size *N_1_* to the information about *π_1_* in a sample of size *wN_2_*. Let the likelihood for *N_1_* be. The weight postulate says this is equivalent to . But the likelihood of *N_2_* must be conserved under a 1-to-1 reparameterization, so $g\left(\pi_{1} \right)^{N_{1} } =g\left(\pi_{1} \right)^{wN_{2} } =g\left(\pi_{2} \right)^{N_{2} }$. For a binomially distributed “failure” (negative animal), $g\left(\pi\right)=1-\pi$. Some algebra yields the relationship $\pi_{2} =1-\left(1-\pi_{1} \right)^{w}$*.* If *π* is the probability of disease, $\varphi=1-\pi$is the probability of being disease-free. From survival analysis theory, it is well-known that being disease-free (“survival”) is related to the cumulative hazard (cumulative force-of-infection in the infection context) via the relationship $\varphi=\exp\left(-\Lambda\right)$ where *Λ* is the cumulative hazard (space does not permit us to give a comprehensive treatment of survival theory; see for example Therneau and Grambsch (2000) for complete developments and references.) Thus we can rewrite $\pi_{2} =1-\left(1-\pi_{1} \right)^{w}$ as $\pi_{2} =1-\exp\left(-\Lambda_{1} w\right)$. Reparameterizing $\mu_{1} =\log\left(\Lambda_{1} \right)$and $\beta=\log\left(w\right)$, we obtain $\pi_{2} =1-\exp\left(-\exp\left(\mu_{1} +\beta\right)\right)$ which can be recognized as the complementary log-log link binary regression model. Here group 1 is the baseline, and the baseline prevalence is given as $\varphi_{1} =\exp\left(-\exp\left(\mu_{1} \right)\right)$; *φ_1_* is the log baseline hazard. *β* is the log hazard ratio, and *w* = exp(*β*) is the hazard ratio. Thus, under the “weight postulate”, if the data are binomially distributed, the nominal weights *w* turn out to be hazard ratios obtainable from the complementary log-log model.

Because of the invariance principle, maximum likelihood estimates of the nominal weights are easy to obtain from the empirical prevalences as: .

When *N* is large and *π* is small, binomial data can be approximated as Poisson data, which was WM’s approach [15]. Under the Poisson approximation, $g\left(\pi\right)=\exp\left(-\pi\right)$. Under the “weight postulate”, we observe $w=\pi_{2} /\pi_{1}$; under the Poisson model, the weight is the Poisson relative risk. The empirical estimate of this relative risk is the WM nominal weight. For small *π*, it is well-known that $\pi\approx-\log\left(1-\pi\right)$, hence the Poisson relative risk is usually very close to the complementary log-log hazard ratio when prevalence is low, which is often the case when attempting to detect disease.

Therneau, T.M. & Grambsch, P.M. (2000) *Modeling Survival Data: Extending the Cox Model*, Springer-Verlag, New York, New York, USA.
