# Supplementary material for: Using Auxiliary Information to Improve Wildlife Disease Surveillance When Infected Animals Are Not Detected: A Bayesian Approach
Source: PLoS One. 2014 Mar 27;9(3):e89843. doi: 10.1371/journal.pone.0089843 (PMC3968012; doi:10.1371/journal.pone.0089843)
Supplement: Software Code S1 — Software Code for Examples. The WinBUGS and SAS code is given here for all of our examples. (DOC) [file pone.0089843.s002.doc]

**Software Code S1**

Note the use of the CUT function; it prevents “feedback” from surveillance data to the log hazard ratio parameter (beta), which recall is based on information from a separate learning data set.

**#PROGRAM 1**

#Posterior distribution with Bayes-Laplace prior

#and the hypothetical surveillance data 0/297.

model{

pi.S~dbeta(1,1)

pos~dbin(pi.S,n)

}

}

#data

list(n=297,pos=0)

**#PROGRAM 2**

#Posterior distribution with Bayes-Laplace prior

#and the hypothetical surveillance data 0/297.

#Now cast in terms of the complementary log-log link

model{

pi.S~dbeta(1,1)

mu.S<-log(-log(1-pi)) #map Bayes-Laplace prior to cloglog scale

predprev<-1-exp(-exp(mu.S))

pos~dbin(predprev,n)

}

}

#data

list(n=297,pos=0)

**#PROGRAM 3**

#Example learning data set of 200 females and 200 males.

#10 positive females and 20 positive males were observed.

#Females are established as the reference baseline group.

#B is the log hazard ratio for males over females

model{

pi.L~dbeta(1,1)

mu.L<-log(-log(1-pi.L))

B.L[1]<-0

B.L[2]~dnorm(0,0.0000001)

for (i in 1:2) {

predprev.L[i]<-1-exp(-exp(mu.L+B.L[sex.L[i]]))

pos.L[i]~dbin(predprev.L[i],n.L[i])

}

}

#data

list(n.L=c(200,200),pos.L=c(10,20), sex.L=c(1,2))

**#PROGRAM 4**

#Example where an learning data set and a surveillance data set are combined.

#As before, the females are established as the reference case.

#In this case, all 297 surveillance samples are assumed to be females.

#Note the use of the cut function, to transfer the learning posterior to the surveillance prior.

model{

pi.L~dbeta(1,1)

mu.L<-log(-log(1-pi.L))

B.L[1]<-0

B.L[2]~dnorm(0,0.0000001)

for (i in 1:2) {

predprev.L[i]<-1-exp(-exp(mu.L+B.L[sex.L[i]]))

pos.L[i]~dbin(predprev.L[i],n.L[i])

}

pi.S~dbeta(1,1)

mu.S<-log(-log(1-pi.S))

B.S[1]<-0

B.S[2]<-cut(B.L[2]) #never gets used in this example

for (i in 1:1) {

predprev.S[i]<-1-exp(-exp(mu.S+B.S[sex.S[i]]))

pos.S[i]~dbin(predprev.S[i],n.S[i])

}

}

#data

list(n.L=c(200,200),pos.L=c(10,20), sex.L=c(1,2),

n.S=c(297),pos.S=c(0), sex.S=c(1)))

**#PROGRAM 5**

#Example where an learning data set and a surveillance data set are combined.

#As before, the females are established as the reference case.

#In this case, all 297 surveillance samples are assumed to be males.

model{

pi.L~dbeta(1,1)

mu.L<-log(-log(1-pi.L))

B.L[1]<-0

B.L[2]~dnorm(0,0.0000001)

for (i in 1:2) {

predprev.L[i]<-1-exp(-exp(mu.L+B.L[sex.L[i]]))

pos.L[i]~dbin(predprev.L[i],n.L[i])

}

pi.S~dbeta(1,1)

mu.S<-log(-log(1-pi.S))

B.S[1]<-0 #never gets used in this example

B.S[2]<-cut(B.L[2])

for (i in 1:1) {

predprev.S[i]<-1-exp(-exp(mu.S+B.S[sex.S[i]]))

pos.S[i]~dbin(predprev.S[i],n.S[i])

}

}

#data

list(n.L=c(200,200),pos.L=c(10,20), sex.L=c(1,2),

n.S=c(297),pos.S=c(0), sex.S=c(2)))

**#PROGRAM 6**

#Analysis of Walsh and Miller's 2010 data. Note reference class has been moved to first position

#in the arrays

model{

pi.L~dbeta(1,1)

mu.L<-log(-log(1-pi.L))

B.L[1]<-0

for (i in 2:8) {

B.L[i]~dnorm(0,0.0000001)

hr.L[i]<-exp(B.L[i])

}

for (i in 1:8) {

predprev.L[i]<-1-exp(-exp(mu.L + B.L[i]))

pos.L[i]~dbin(predprev.L[i],n.L[i])

}

}

#data

list(n.L=c(10146,111,125,1300,5782,645,1329,999),pos.L=c(313,40,40,77,104,9,11,1))

**#PROGRAM 7**

#Synthetic surveillance example using Walsh and Miller's learning set.

model{

pi.L~dbeta(1,1)

mu.L<-log(-log(1-pi.L))

B.L[1]<-0

for (i in 2:8) {

B.L[i]~dnorm(0,0.0000001)

}

for (i in 1:8) {

predprev.L[i]<-1-exp(-exp(mu.L + B.L[class.L[i]]))

pos.L[i]~dbin(predprev.L[i],n.L[i])

}

pi.S~dbeta(1,1)

mu.S<-log(-log(1-pi.S))

B.S[1]<-0

for (i in 2:8) {

B.S[i]<-cut(B.L[i])

}

for (i in 1:3) {

predprev.S[i]<-1-exp(-exp(mu.S + B.L[class.S[i]]))

pos.S[i]~dbin(predprev.S[i],n.S[i])

}

}

#data

list(n.L=c(10146,111,125,1300,5782,645,1329,999),pos.L=c(313,40,40,77,104,9,11,1),

class.L=c(1,2,3,4,5,6,7,8),

class.S=c(2,3,4),n.S=c(10,10,23), pos.S=c(0,0,0))

**/*PROGRAM 8**

SAS Code to obtain MLEs of our nominal and Walsh and Miller’s weights */

data a;

input grp c n;

cards;

1 40 111

2 40 125

3 77 1300

0 313 10046

4 104 5782

5 9 645

6 11 1392

7 1 999

;

run;

data a;

set a;

array g {*} g1-g7;

do i = 1 to 7;

g[i] = grp eq i;

end;

learn = 1;

proc genmod;

model c / n = g1-g7 learn/error=binomial link=cloglog noint;

estimate 'g1' g1 1/exp;

estimate 'g2' g2 1/exp;

estimate 'g3' g3 1/exp;

estimate 'g4' g4 1/exp;

estimate 'g5' g5 1/exp;

estimate 'g6' g6 1/exp;

estimate 'g7' g7 1/exp;

run;

proc genmod;

model c / n = g1-g7 learn/error=poisson link=log noint;

estimate 'g1' g1 1/exp;

estimate 'g2' g2 1/exp;

estimate 'g3' g3 1/exp;

estimate 'g4' g4 1/exp;

estimate 'g5' g5 1/exp;

estimate 'g6' g6 1/exp;

estimate 'g7' g7 1/exp;

run;
